# Inequality in hospital bed supply and utilization across regions and departments: evidence from China

**DOI:** 10.1186/s12962-026-00827-9

**Published:** 2026-09-24

**Authors:** Yuheng Zhang, Jiyun Cui, Juan Li, Yan Song, Yin Shi

**Affiliations:** School of Health Care Security, Shandong First Medical University, Jinan, 250117 China

**Keywords:** Hospital beds, Bed supply, Bed turnover rate, Regional disparities, Interdepartmental disparities, China

## Abstract

**Background:**

Adequate hospital bed capacity is fundamental to health-care accessibility. However, increasing beds per capita may mask inequalities across regions and departments, not only in supply but also in how available capacity and utilization patterns vary across regions and departments. This study examined spatiotemporal patterns and inequalities in hospital bed supply and utilization across regions and departments in China from 2009 to 2024 to inform more targeted bed planning and resource allocation.

**Methods:**

Using data on hospital beds, inpatient discharges, and population for 15 hospital departments across 31 provincial-level regions in mainland China, we measured supply as beds per 10,000 population and utilization as the bed turnover rate. Spatial analysis, kernel density estimation, and Markov chains characterized distributional patterns and dynamics, while GE(1) measured interprovincial inequality within departments and interdepartmental inequality within provinces.

**Results:**

National bed supply increased from 23.56 to 57.74 beds per 10,000 population, substantially outpacing growth in the bed turnover rate, which rose from 27.09 to 31.25. Markov analysis showed a clear upward shift in bed supply. Provinces in the lower-middle and upper-middle states moved to the next-higher state with probabilities of 0.71 and 0.51, respectively, while the high state had a persistence probability of 0.98. The low and high bed-turnover states were relatively stable, but no consistent upward shift emerged. GE(1) showed that interprovincial inequality in overall bed supply declined from 0.03 to 0.01, whereas inequality in bed turnover rates remained at approximately 0.01. However, bed-supply inequality increased in obstetrics and gynecology, infectious diseases, and traditional medicine. Within provinces, interdepartmental inequality in bed supply declined from 0.55 to 0.49 but remained substantially greater than that in bed turnover rates.

**Conclusions:**

Expansion of total bed capacity and the narrowing of interprovincial disparities did not eliminate persistent disparities across departments. Hospital-bed planning should manage new capacity and reallocate existing beds based on region- and department-specific assessments of supply, utilization, and need.

## Introduction

Hospital beds are a fundamental component of the physical infrastructure of healthcare systems [1]. Insufficient bed capacity may create barriers to access, contribute to overcrowding and delays in admission, and increase pressure on inpatient services. Conversely, excess capacity, or capacity that is not allocated according to service needs, may result in the inefficient use of scarce resources [2]. The appropriate allocation of hospital beds has long been a common challenge for healthcare systems worldwide [3], affecting countries such as the United States, Japan, Iran, and China to varying degrees [4–7]. In China, government investment in health care has continued to increase since the launch of a new round of healthcare reform in 2009 [8], accompanied by a substantial expansion in hospital bed capacity. Still, the distribution of beds remains uneven—both geographically across regions and structurally across clinical departments [9, 10]. Moreover, these imbalances are evident in both supply (e.g., beds per capita) and utilization (e.g., bed turnover rate), suggesting that a high level of bed supply does not necessarily correspond to higher levels of bed utilization [11, 12]. Therefore, systematically assessing inequalities in hospital bed supply and utilization across both regions and clinical departments is important for identifying potential differences between supply and utilization patterns and informing strategies to optimize health resource allocation.

Existing studies have made important contributions to understanding interregional inequality in hospital bed supply. By analyzing total bed numbers or beds per capita across different areas, researchers have documented regional disparities in bed supply and their changes over time [13–15], and have examined the spatial distribution of bed resources and residents’ access to inpatient care [16, 17]. Some studies have used measures such as the Gini coefficient and Theil index to quantify inequality in the distribution of hospitals and hospital beds and to investigate its spatial sources [18, 19]. Others have applied measures such as the health resource density index to analyze bed allocation in terms of its spatiotemporal evolution and determinants [20–22], showing that disparities exist not only between regions but also within them. However, most studies focus on total bed numbers at the regional level, effectively treating beds across different clinical departments as homogeneous resources and paying limited attention to structural differences between departments within regions. The limited department-level literature mainly focuses on a single department, such as intensive care, psychiatry, oncology, or rehabilitation [23–26]. It largely examines bed supply, making it difficult to compare supply and utilization systematically across departments within the same region. On the other hand, studies of bed utilization typically use bed turnover rates, occupancy rates, and average length of stay, but most merely describe utilization patterns without further examining how they correspond to bed supply [27–29]. To assess hospital bed allocation more comprehensively, it is necessary to examine disparities across regions and clinical departments and to assess bed supply alongside utilization.

This study systematically examines changes in inequality in hospital bed supply and utilization across regions and clinical departments in China from 2009 to 2024. The analysis addresses three questions. First, how have the spatiotemporal patterns of bed supply and utilization changed, and how do they differ across regions and departments? Second, how have the distributions of the two indicators evolved, and what patterns of state transition have emerged? Third, how has interprovincial inequality in bed supply and utilization changed within each department, and what patterns of inequality exist across departments within each province? To answer these questions, we construct a province–department–year dataset and use descriptive statistics, spatial analysis, kernel density estimation, Markov chain analysis, and the generalized entropy index to examine spatiotemporal patterns, distributional dynamics, and changes in inequality. The findings may inform a shift in hospital bed planning beyond aggregate capacity toward region- and department-specific adjustments, while supporting department-specific service capacity development and more adaptive resource allocation.

## Methods

### Data sources

**Fig. 1:**
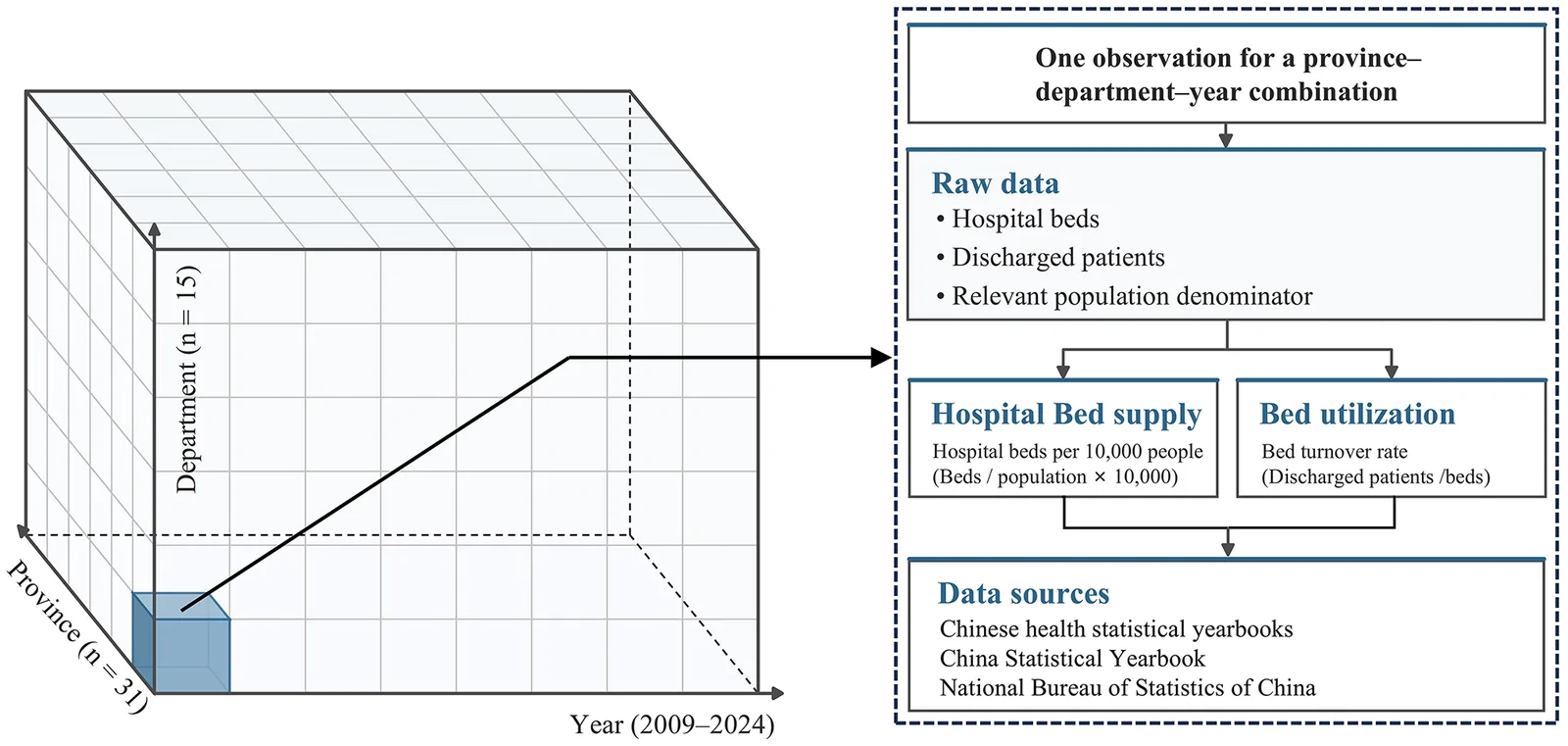
Province–department–year data framework

This study assessed hospital bed allocation from two complementary dimensions: bed supply and bed utilization. Bed supply was measured by the number of hospital beds per 10,000 population, a widely used indicator of inpatient service availability and hospital service capacity [30]. Bed utilization was measured by the bed turnover rate, defined as the annual number of discharged patients divided by the number of beds in the corresponding department. This indicator reflects the number of patients served by each bed during a year and is commonly used to assess bed utilization and service activity [31]. To simultaneously examine the regional and departmental dimensions of hospital bed allocation, we constructed a three-dimensional panel dataset organized by province, department, and year (Fig. 1). It covered 31 provincial-level regions in mainland China (excluding Hong Kong SAR, Macao SAR, and Taiwan), 15 major clinical departments, and 16 years from 2009 to 2024. This structure yielded 7,440 potential observations per indicator (31 × 15 × 16). This three-dimensional structure allows us to capture both interprovincial heterogeneity and intra-provincial cross-departmental heterogeneity in bed supply and utilization over time—a distinction that is not feasible with conventional two-dimensional region–year panel data, which treat all beds within a region as aggregated.

The 15 departments were defined primarily according to the *List of Medical Diagnosis and Treatment Categories of Medical Institutions*. To ensure analytical consistency, we combined several related departments: Preventive Medicine and General Medicine were grouped as Preventive and General Medicine; Ophthalmology, Otorhinolaryngology, and Stomatology were grouped as ENT–Ophthalmology–Stomatology; Traditional Chinese Medicine and Ethnomedicine were grouped as Traditional Medicine; Tuberculosis was incorporated into Infectious Diseases; and other smaller or less frequently reported departments (including but not limited to Occupational Medicine, Critical Care Medicine, and Emergency Medicine) were grouped as Other Departments (abbreviations for all departments are presented in Table 1).

Department-level data on hospital beds and discharged patients were obtained from the *China Health Statistical Yearbook* (2009–2012), the *China Health and Family Planning Statistical Yearbook* (2013–2017), and the *China Health Statistics Yearbook* (2018–2025). Provincial resident population data were obtained from the *China Population and Employment Statistical Yearbook* (2009–2025). For most departments, the total resident population of each province was used as the denominator. Because Pediatrics and Obstetrics and Gynecology serve populations with distinct age and sex characteristics, the population aged 0–14 years was used as the denominator for Pediatrics, while the female population aged 15 years and older was used for Obstetrics and Gynecology. These population data were obtained from the *China Population and Employment Statistical Yearbook*.

**Table 1:** Abbreviations for department names

Department Type	Abbreviation
Preventive Medicine Dept. & General Medicine Dept.	PMD & GMD
Internal Medicine Dept.	IM Dept.
Surgery Dept.	Surg Dept.
Pediatrics Dept.	Peds Dept.
Gynecology Dept. & Obstetrics Dept.	OB/GYN Dept.
E.N.T. Dept. & Ophthalmology Dept. & Stomatology Dept.	ENT-Ophth-Stom
Dermatology Dept.	Derm Dept.
Aesthetic Medicine Dept.	AMD
Psychiatry Dept.	Psych Dept.
Infectious Diseases Dept. & Tuberculosis Dept.	ID & TB
Oncology Dept.	Onc Dept.
Rehabilitation Medicine Dept.	Rehab Dept.
Traditional Chinese Medicine (TCM) Dept. & Ethnomedicine Dept.	TCM & Emd.
Integrated TCM & Western Medicine Dept.	ITCWM Dept.
Other Depts. (e.g., Occupational Medicine, Critical Care Medicine, and Emergency Medicine)	Other Depts.

Missing values in a small number of province–department–year observations were handled as follows. Consecutive multi-year gaps in both bed and discharge counts—including AMD in Tibet (2009–2015), Rehabilitation Medicine in Tibet (2009–2013), and AMD in Qinghai (2009–2016)—and in discharge counts—including Integrated Traditional Chinese and Western Medicine in Tibet (2010–2012) and Psychiatry in Tibet (2009–2010)—were assigned a value of zero, as the values at first appearance were extremely small, indicating that these departments had either not yet been established or were effectively inactive during the initial years. Isolated single-year gaps were imputed using the mean of adjacent years [32], including four bed count observations in Tibet (Psychiatry in 2010, Integrated Traditional Chinese and Western Medicine in 2010 and 2013, and Oncology in 2012), two discharge count observations in Tibet (Dermatology in 2010 and Oncology in 2012), and two AMD discharge-count observations in Inner Mongolia and Ningxia in 2011. In addition, the bed turnover rate for AMD in Tianjin was identified as an outlier: the rate was 4.11 in 2021 but surged to 1,060.49 in 2024, markedly outside its historical range; this observation was therefore excluded from the relevant analyses.

### Kernel density estimation

Kernel density estimation is a non-parametric statistical method used to estimate the probability density function of a random variable by capturing the continuity and overall trend of the sample distribution [33]. Kernel density estimation (KDE) was used to examine changes in the distribution of hospital beds per 10,000 population and bed turnover rates across provinces. The density function was estimated as:

1 $$f(x)=\frac{1}{{nh}}\sum\limits_{{i=1}}^{n} k \left( {\frac{{x - {X_i}}}{h}} \right)$$

where *n* is the number of observations, ${X_i}$ is the observed value, *h* is the bandwidth, and *k( · )* is the kernel function. A Gaussian kernel was used:

2 $$k(u)=\frac{1}{{\sqrt {2\pi } }}\exp \left( { - \frac{{{u^2}}}{2}} \right)$$

3 $$u=\frac{{x - {X_i}}}{h}$$

The bandwidth was selected using Silverman’s rule of thumb:

4 $$h=0.9\hbox{min} \left( {s,\frac{{{\mathrm{IQR}}}}{{1.34}}} \right){n^{ - 1/5}}$$

where *s* is the sample standard deviation and IQR is the interquartile range. KDE was conducted separately for each indicator, department, and selected year to compare changes in the location, dispersion, and shape of provincial distributions over time. All KDE analyses were performed using EViews 12.

### Markov chain analysis

Markov chain analysis was used to examine changes in hospital bed supply and utilization across provinces over time. As a discretized Markov process, it features three key properties: the memoryless property (or lack of after-effect), discrete time and state spaces, and the independence of future states from historical trajectories [34]. For each indicator and department, the provincial observations were classified into four states according to the quartiles of the distribution in the initial year: low (State 1, at or below the 25th percentile), lower-middle (State 2, between the 25th and 50th percentiles), upper-middle (State 3, between the 50th and 75th percentiles), and high (State 4, above the 75th percentile). For each one-year transition from t to t + 1, observations in both years were classified using the thresholds derived from the initial year t. The thresholds were recalculated for each subsequent transition period rather than fixed throughout 2009–2024. Let the random variable *Xt* = *j* denote that the system is in state *j* at time *t*, as expressed in Formula (5).

5 $$P\{X_t=j\mid X_{t-1}=i,X_{t-2}=i_{t-2},\ldots,X_0=i_0\}=P\{X_t=j\mid X_{t-1}=i\}=P_{ij}$$

Assume that ${P_{ij}}$ represents the transition probability of departmental bed resource allocation from state *i* in year t to state j in year t + 1. ${n_i}$ denotes the overall number of instances when bed resources were in allocation state *i* throughout the sample period and ${n_{ij}}$ represents the count of occurrences where bed resources transitioned from state *i* to state *j*. Accordingly, ${P_{ij}}$ can be estimated using the Maximum Likelihood Estimation method, as expressed in Formula (6).

6 $${P_{ij}}={{{n_{ij}}} \mathord{\left/ {\vphantom {{{n_{ij}}} {{n_i}}}} \right. \kern-0pt} {{n_i}}}$$

### Generalized entropy index

The generalized entropy (*GE*) index was introduced by Shorrocks (1980) based on information theory [35]. It comprises a family of inequality measures satisfying three axioms: mean independence, the transfer principle, and additive decomposability. Its general form is:

7 $$GE(\alpha)=\frac{1}{\alpha(\alpha-1)}\left[\frac{1}{n}\sum_{i=1}^{n}\left(\frac{y_i}{\bar{y}}\right)^{\alpha}-1\right],\quad\alpha\neq0,1$$

Here, *n* denotes the number of provinces (or hospital departments), *y*_*i*_ is the observed value for province (or department) *i*, such as the number of hospital beds per 10,000 population, and $\bar {y}$ is the mean across all provinces (or departments). The parameter *α* determines the index’s sensitivity to different parts of the distribution. Smaller values of *α* place greater weight on changes at the lower end of the distribution, whereas larger values place greater weight on changes at the upper end. Two widely used special cases of the generalized entropy index are *GE*(0) and *GE*(1). *GE*(0), known as the mean log deviation (MLD) or Theil *L* index, is defined in Eq. (8). *GE*(1), also known as the Theil *T* index, is defined in Eq. (9) [36]. Compared with *GE*(0) and other members of the generalized entropy family, *GE*(1) is not excessively sensitive to extreme values, thereby reducing the potential for anomalous fluctuations in individual provinces to distort the overall pattern. Moreover, *GE*(1) is among the most widely used generalized entropy measures and is well established in the literature on healthcare accessibility and equity [37–39]. Accordingly, this study uses *GE*(1) to quantify disparities in hospital bed supply and bed utilization across provinces and hospital departments. It should be noted that the Generalized Entropy index GE(1) relies on logarithmic transformation, which requires special handling of zero-valued observations in direct numerical computation [40]. To circumvent this limitation, we adopted the approach proposed by Manero (2017) [41] and replaced all zero values with a negligibly small positive constant (10⁻¹⁰), thereby ensuring the computational feasibility of the index. These zero values were retained in descriptive analyses and were replaced only during GE(1) calculation because of the logarithmic transformation requirement.

8 $$GE(0)=\frac{1}{n}\sum\limits_{{i=1}}^{n} {\ln \left( {\frac{{\bar {y}}}{{{y_i}}}} \right)} $$

9 $$GE(1)=\frac{1}{n}\sum\limits_{{i=1}}^{n} {\frac{{{y_i}}}{{\bar {y}}}} \ln \left( {\frac{{{y_i}}}{{\bar {y}}}} \right)$$

## Results

### Spatiotemporal patterns of hospital bed supply and utilization

#### Changes in hospital bed supply and utilization by department

**Fig. 2:**
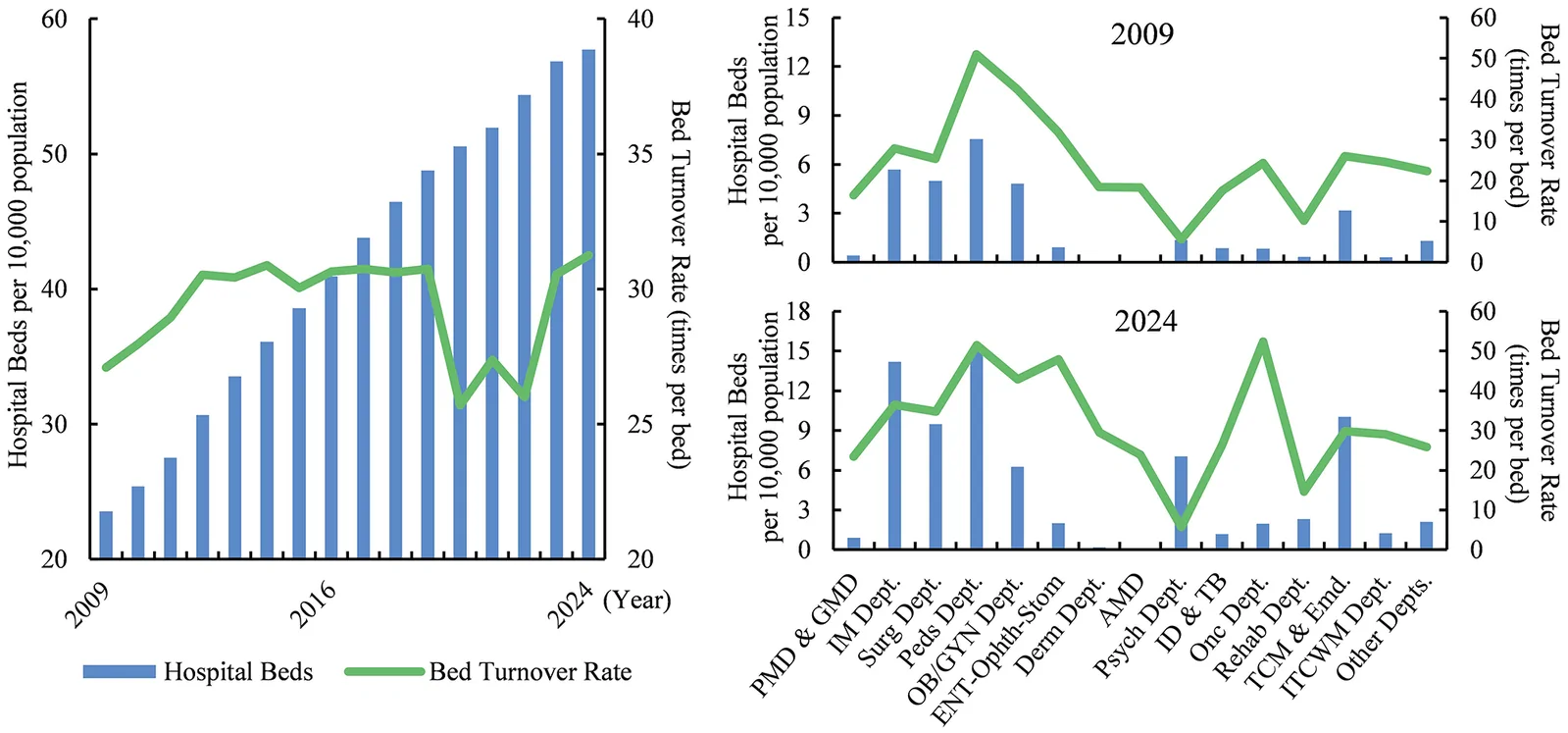
Temporal and departmental patterns of hospital bed supply and utilization

Figure 2 illustrates temporal trends in hospital bed supply and utilization in China, alongside variations in bed distribution across clinical departments. From 2009 to 2024, national bed density increased by 145.08%, from 23.56 to 57.74 beds per 10,000 population, whereas the bed turnover rate increased by 15.36%, from 27.09 to 31.25. Compared with the sustained expansion in bed capacity, changes in bed utilization were relatively modest. The turnover rate remained around 30 for most of the study period, declined to 25.71 in 2020, and subsequently recovered.

Departmental patterns also differed markedly. In 2024, pediatrics and internal medicine had the highest bed-density values, at 15.11 and 14.18, respectively, followed by traditional medicine at 10.02 and surgery at 9.50. Bed supply also increased substantially in psychiatry and rehabilitation medicine, although their turnover rates remained relatively low at 5.72 and 14.61, respectively. By contrast, oncology and pediatrics recorded the highest turnover rates, at 52.40 and 51.46. Overall, bed expansion and utilization did not evolve in parallel across departments. Bed supply continued to increase overall, whereas changes in utilization were more limited and varied across departments.

#### Changes in hospital bed supply and utilization by region

**Fig. 3:**
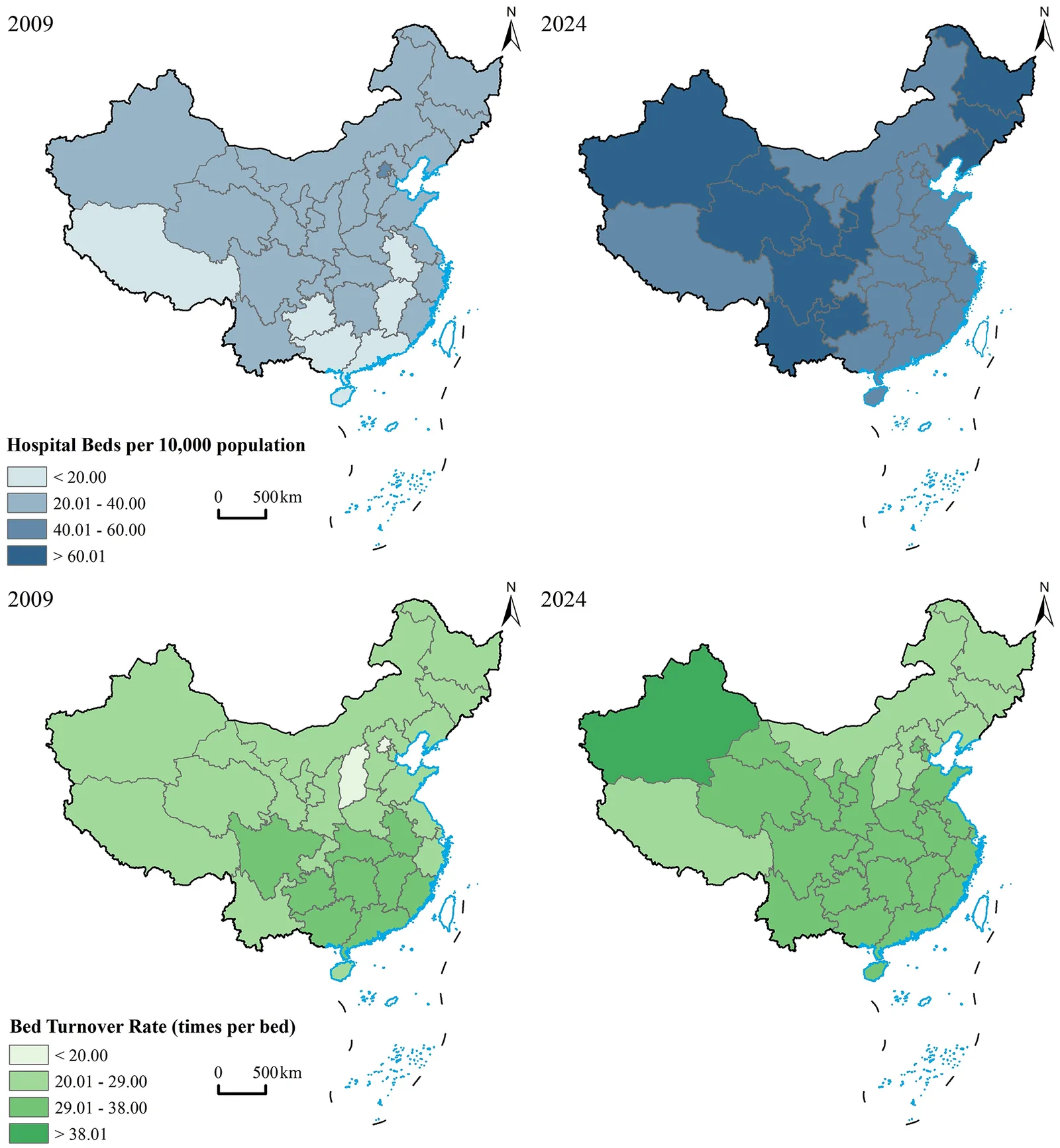
Spatial distribution of hospital bed supply and utilization in China

Hospital bed supply was generally low in 2009. Most provinces had 20.01–40.00 beds per 10,000 population, and Beijing was the only province with more than 40. Anhui, Guizhou, Guangxi, and Tibet, located in central and western China, had fewer than 20 beds per 10,000 population. By 2024, bed density had risen across the country, with most provinces exceeding 40 beds per 10,000 population. The increase was more pronounced in western and northeastern China, while eastern coastal and central provinces were mainly concentrated within the range of 40.01–60.00 (Fig. 3).

Bed turnover increased in most provinces, although the pattern varied across regions. The rates in Beijing and Tianjin in eastern China rose from 19.03 to 22.14 in 2009 to 35.46 and 37.20 in 2024, whereas Hebei and Fujian declined slightly. Henan and Hubei in central China recorded increases, Anhui changed little, and Jiangxi fell from 32.37 to 29.40. Several western provinces also showed marked growth. Xinjiang reached 38.02 in 2024, the highest value nationwide, while Tibet remained at a relatively low level. Turnover also increased in Liaoning, Jilin, and Heilongjiang, but remained below 29 in all three northeastern provinces in 2024.

#### Changes in hospital bed supply and utilization by region and department

**Fig. 4:**
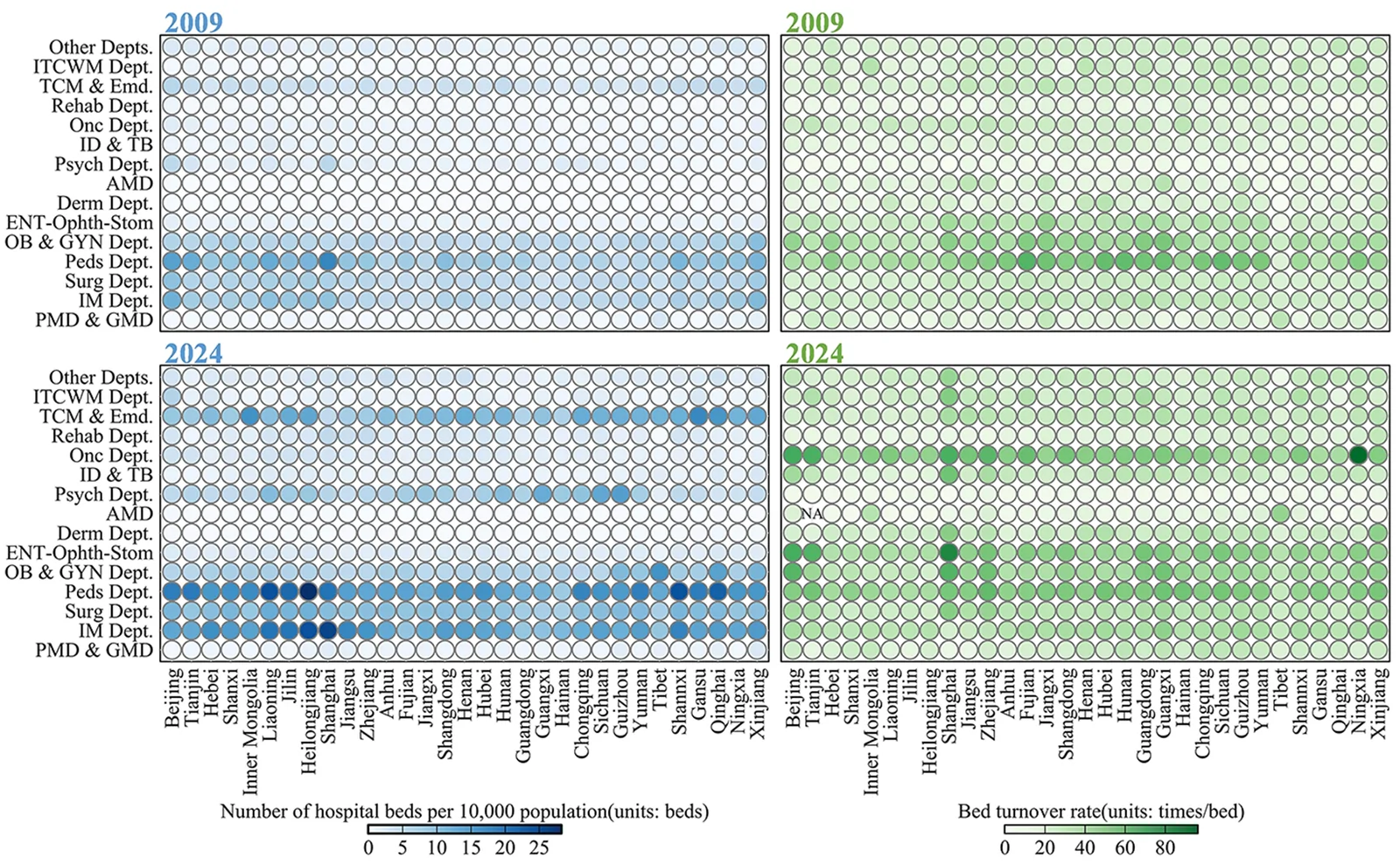
Hospital bed supply and utilization across provinces and departments. Note: NA indicates that the 2024 bed turnover observation for medical aesthetics in Tianjin was treated as an outlier and excluded from the analysis

Figure 4 compares changes in hospital bed supply and utilization across provinces and departments between 2009 and 2024. Growth in bed density was concentrated in internal medicine, surgery, pediatrics, and traditional medicine, although the geographic pattern differed by department. Internal medicine and surgical beds increased markedly in the northeastern provinces of Liaoning, Jilin, and Heilongjiang, as well as in eastern provincial-level regions such as Shanghai and Jiangsu. Rapid growth in pediatric beds was observed not only in Northeast China but also in the western provinces of Sichuan, Guizhou, Yunnan, and Qinghai. Growth in traditional medicine beds was concentrated in western China, particularly in Gansu, Ningxia, and Inner Mongolia. Psychiatric bed growth was most apparent in the southwestern provincial-level regions of Guangxi, Chongqing, Sichuan, and Guizhou. Bed density in medical aesthetics and dermatology remained low in most provinces.

Oncology showed the most pronounced change in bed turnover. In 2009, oncology turnover was relatively low in most provinces. By 2024, the increase was most pronounced in Ningxia, while Beijing, Tianjin, and Shanghai in eastern China had also moved into higher categories. Turnover in ENT–ophthalmology–stomatology also rose noticeably, with high values concentrated mainly in Beijing, Tianjin, and Shanghai. Changes in bed supply and utilization did not always move in the same direction. Psychiatric beds increased rapidly in western provinces such as Guangxi, Sichuan, and Guizhou, but turnover remained low. A similar divergence between bed growth and turnover was observed for rehabilitation medicine in several provinces.

### Distribution dynamics and evolution trends

#### Kernel density estimation

**Fig. 5:**
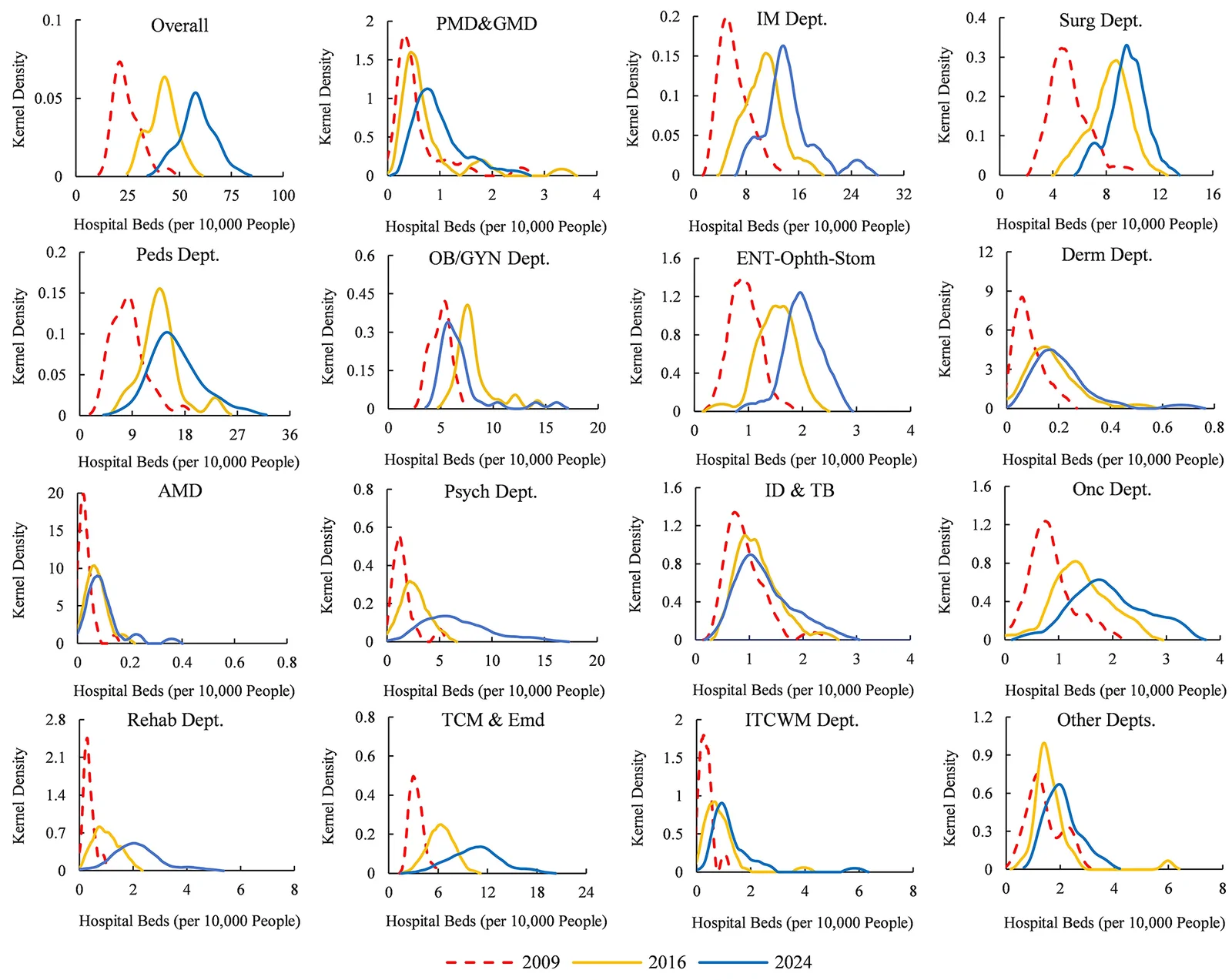
Evolution of bed supply distributions by department

From 2009 to 2024, the distribution of hospital beds per 10,000 population shifted to the right, with lower peaks and a progressively wider range. Bed supply therefore increased across provinces, although interprovincial differences persisted. The largest shifts were observed in internal medicine, surgery, and traditional medicine. For most departments, peak heights declined, the curves widened, and the right tails lengthened, reflecting greater dispersion in bed supply across provinces. Preventive and general medicine, obstetrics and gynecology, and dermatology showed clear right-skewed distributions with broader ranges. The aggregate distribution remained predominantly unimodal, whereas preventive and general medicine, internal medicine, and integrated traditional Chinese and Western medicine changed from unimodal to bimodal patterns, suggesting some separation among provinces (Fig. 5).

**Fig. 6:**
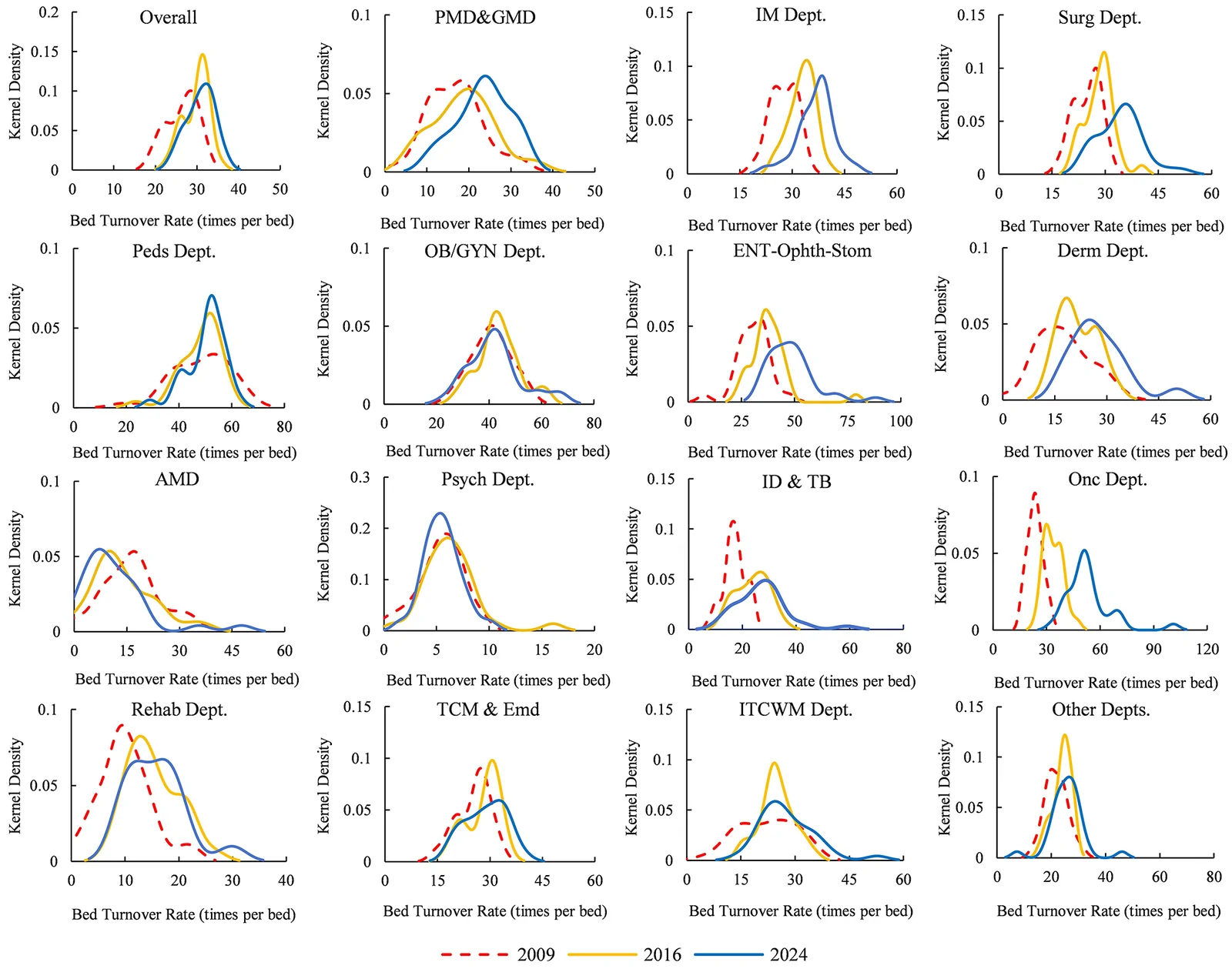
Evolution of bed turnover distributions by department

The overall distribution of bed turnover rates also shifted to the right, but the movement was smaller than that observed for bed supply. Oncology showed the most pronounced rightward shift. Its distribution moved progressively towards higher values, and the 2024 curve widened with a longer right tail, suggesting greater variation across provinces. Internal medicine, surgery, ENT–ophthalmology–stomatology, and infectious diseases also shifted rightward. In some departments, the peaks became lower, the tails lengthened, and the distributions changed from unimodal to bimodal or multimodal patterns. Pediatrics and obstetrics and gynecology remained in relatively high turnover ranges, whereas psychiatry stayed concentrated at low levels with little change over the study period. Compared with the broad expansion of bed supply, increases in turnover were concentrated in a smaller group of departments, particularly oncology, internal medicine, and infectious diseases (Fig. 6).

#### Markov chain analysis

Table 2 shows a clear upward transition in bed supply. Provinces in the lower-middle and upper-middle states transitioned to the next-higher state with probabilities of 0.71 and 0.51, respectively, while the probability of remaining in the high state reached 0.98. For bed turnover rates, the low and high states were relatively stable, with persistence probabilities of 0.73 and 0.79, respectively. Provinces in the upper-middle state had similar probabilities of remaining in that state (0.43) and transitioning to the high state (0.38).

At the department level, the high bed-supply state was strongly persistent: the probability of remaining in this state was at least 0.90 in 12 of the 15 departments. The traditional medicine department showed the strongest upward transition, with probabilities of 0.52 for moving from the lower-middle to the upper-middle state and 0.59 for moving from the upper-middle to the high state. For most department–state combinations, bed turnover rates were most likely to remain in the same state, although transition patterns varied across departments. The probabilities of remaining in the high state were 0.90 for internal medicine and 0.88 for oncology. In oncology, the probability of transitioning from the lower-middle to the upper-middle state was 0.48, exceeding the probability of remaining in the lower-middle state (0.38) (Tables 3 and 4).

**Table 2:** Markov transition probability matrices for hospital beds per 10,000 population and bed turnover rate (t = 1)

t = 1	1	2	3	4		1	2	3	4
	Hospital Beds per 10,000 population					Bed Turnover Rate			
1	0.64	0.35	0.01	0.00		0.73	0.23	0.02	0.03
2	0.00	0.27	0.71	0.03		0.11	0.58	0.23	0.08
3	0.00	0.01	0.48	0.51		0.03	0.15	0.43	0.38
4	0.00	0.00	0.02	0.98		0.04	0.05	0.12	0.79

Note: State 1 denotes low, State 2 lower-middle, State 3 upper-middle, and State 4 high. t = 1 denotes a one-year transition. The same applies to the following tables

**Table 3:** Markov transition probability matrices for hospital beds per 10,000 population across 15 departments (t = 1)

t = 1	1	2	3	4		1	2	3	4		1	2	3	4
	PMD & GMD					IM Dept.					Surg Dept.			
1	0.56	0.31	0.11	0.03		0.74	0.26	0.00	0.00		0.78	0.18	0.04	0.00
2	0.16	0.50	0.29	0.05		0.00	0.48	0.51	0.01		0.02	0.53	0.38	0.08
3	0.08	0.15	0.55	0.22		0.00	0.04	0.56	0.40		0.00	0.05	0.67	0.28
4	0.00	0.02	0.11	0.87		0.00	0.00	0.01	0.99		0.00	0.01	0.02	0.97
	Peds Dept.					OB/GYN Dept.					ENT-Ophth-Stom			
1	0.69	0.25	0.03	0.03		0.69	0.29	0.01	0.01		0.60	0.38	0.03	0.00
2	0.11	0.45	0.34	0.10		0.11	0.56	0.30	0.03		0.04	0.55	0.38	0.03
3	0.05	0.10	0.39	0.46		0.01	0.17	0.50	0.33		0.00	0.02	0.59	0.39
4	0.02	0.01	0.03	0.94		0.00	0.00	0.10	0.90		0.00	0.00	0.04	0.96
	Derm Dept.					AMD					Psych Dept.			
1	0.74	0.22	0.04	0.00		0.75	0.21	0.03	0.01		0.64	0.36	0.00	0.00
2	0.12	0.68	0.21	0.00		0.20	0.57	0.22	0.02		0.01	0.62	0.38	0.00
3	0.00	0.13	0.69	0.18		0.04	0.17	0.57	0.23		0.00	0.01	0.65	0.34
4	0.00	0.00	0.10	0.90		0.01	0.05	0.12	0.82		0.00	0.00	0.01	0.99
	ID & TB					Onc Dept.					Rehab Dept.			
1	0.80	0.18	0.02	0.00		0.71	0.28	0.01	0.00		0.65	0.28	0.07	0.00
2	0.08	0.70	0.23	0.00		0.02	0.71	0.28	0.00		0.03	0.60	0.37	0.01
3	0.02	0.08	0.73	0.17		0.00	0.01	0.68	0.32		0.00	0.03	0.47	0.51
4	0.00	0.00	0.06	0.94		0.00	0.00	0.02	0.98		0.00	0.00	0.04	0.96
	TCM & Emd.					ITCWM Dept.					Other Depts.			
1	0.63	0.33	0.03	0.01		0.70	0.28	0.03	0.00		0.61	0.28	0.10	0.01
2	0.02	0.43	0.52	0.03		0.03	0.67	0.29	0.01		0.14	0.29	0.49	0.08
3	0.00	0.00	0.41	0.59		0.01	0.04	0.62	0.33		0.08	0.09	0.53	0.30
4	0.00	0.00	0.03	0.97		0.00	0.00	0.01	0.99		0.06	0.03	0.05	0.87

**Table 4:** Markov transition probability matrices for bed turnover rate across 15 departments (t = 1)

t = 1	1	2	3	4		1	2	3	4		1	2	3	4
	PMD & GMD					IM Dept.					Surg Dept.			
1	0.64	0.28	0.05	0.03		0.75	0.18	0.03	0.03		0.77	0.23	0.01	0.00
2	0.19	0.53	0.21	0.08		0.09	0.55	0.28	0.08		0.07	0.63	0.23	0.07
3	0.05	0.14	0.51	0.30		0.04	0.08	0.53	0.35		0.01	0.07	0.46	0.47
4	0.02	0.06	0.21	0.71		0.02	0.02	0.07	0.90		0.01	0.02	0.10	0.87
	Peds Dept.					OB/GYN Dept.					ENT-Ophth-Stom			
1	0.76	0.15	0.06	0.03		0.82	0.13	0.05	0.00		0.75	0.19	0.06	0.00
2	0.22	0.48	0.15	0.16		0.28	0.38	0.33	0.01		0.09	0.61	0.25	0.05
3	0.11	0.21	0.38	0.31		0.08	0.15	0.54	0.23		0.02	0.09	0.58	0.32
4	0.06	0.10	0.09	0.76		0.00	0.01	0.14	0.85		0.00	0.02	0.07	0.91
	Derm Dept.					AMD					Psych Dept.			
1	0.61	0.31	0.07	0.02		0.80	0.17	0.02	0.01		0.79	0.19	0.01	0.01
2	0.14	0.54	0.28	0.03		0.41	0.31	0.18	0.11		0.10	0.61	0.29	0.00
3	0.03	0.16	0.49	0.33		0.14	0.38	0.38	0.10		0.01	0.18	0.62	0.19
4	0.02	0.05	0.16	0.77		0.09	0.03	0.34	0.54		0.01	0.04	0.09	0.87
	ID & TB					Onc Dept.					Rehab Dept.			
1	0.78	0.19	0.03	0.01		0.53	0.39	0.06	0.02		0.74	0.20	0.04	0.02
2	0.11	0.53	0.23	0.13		0.08	0.38	0.48	0.06		0.10	0.63	0.25	0.03
3	0.05	0.07	0.48	0.41		0.03	0.09	0.45	0.43		0.00	0.15	0.63	0.22
4	0.00	0.08	0.10	0.83		0.00	0.01	0.11	0.88		0.00	0.01	0.11	0.88
	TCM & Emd.					ITCWM Dept.					Other Depts.			
1	0.83	0.17	0.00	0.00		0.68	0.23	0.08	0.00		0.68	0.21	0.08	0.03
2	0.06	0.63	0.27	0.04		0.14	0.45	0.33	0.08		0.15	0.47	0.29	0.09
3	0.00	0.20	0.40	0.40		0.02	0.25	0.39	0.34		0.04	0.18	0.47	0.32
4	0.01	0.02	0.11	0.86		0.02	0.06	0.23	0.70		0.02	0.09	0.19	0.70

### Inequality in hospital bed supply and utilization

#### Interprovincial inequality by department

The GE(1) results showed an overall reduction in interprovincial inequality in hospital bed supply. The overall index declined from 0.03 in 2009 to 0.01 in 2024. Across departments, the index decreased in most categories, with larger reductions in preventive and general medicine, psychiatry, and rehabilitation medicine, but modest increases in obstetrics and gynecology, infectious diseases, and traditional medicine. In 2024, medical aesthetics and integrated traditional Chinese and Western medicine had two of the largest indices, at 0.20 and 0.19, respectively, indicating substantial heterogeneity in interprovincial bed-supply disparities across departments (Table 5).

Interprovincial inequality in bed turnover rates changed little at the aggregate level, with the overall index remaining close to 0.01 throughout 2009–2024. The indices for preventive and general medicine, dermatology, and rehabilitation medicine declined, whereas those for internal medicine, surgery, and traditional medicine changed little. Modest increases occurred in obstetrics and gynecology, infectious diseases, and oncology (Table 6).

**Table 5:** Interprovincial inequality in hospital bed supply by department

Departments	2009	2010	2011	2012	2013	2014	2015	2016	2017	2018	2019	2020	2021	2022	2023	2024
Overall	0.03	0.03	0.02	0.02	0.02	0.01	0.01	0.01	0.01	0.01	0.01	0.01	0.01	0.01	0.01	0.01
PMD & GMD	0.28	0.34	0.37	0.28	0.31	0.35	0.30	0.25	0.23	0.17	0.13	0.11	0.10	0.10	0.11	0.09
IM Dept.	0.06	0.05	0.04	0.04	0.03	0.03	0.03	0.03	0.03	0.03	0.03	0.03	0.03	0.03	0.03	0.03
Surg Dept.	0.03	0.03	0.02	0.02	0.02	0.02	0.01	0.01	0.01	0.01	0.01	0.01	0.01	0.01	0.01	0.01
Peds Dept.	0.06	0.05	0.04	0.04	0.03	0.03	0.03	0.03	0.03	0.03	0.03	0.03	0.03	0.03	0.03	0.03
OB/GYN Dept.	0.03	0.02	0.02	0.02	0.02	0.02	0.03	0.03	0.03	0.03	0.03	0.04	0.04	0.05	0.06	0.06
ENT-Ophth-Stom	0.04	0.04	0.03	0.03	0.02	0.02	0.02	0.03	0.03	0.02	0.02	0.02	0.02	0.02	0.01	0.01
Derm Dept.	0.18	0.17	0.15	0.15	0.14	0.14	0.15	0.16	0.16	0.16	0.15	0.15	0.13	0.12	0.11	0.13
AMD	0.35	0.37	0.31	0.25	0.23	0.21	0.21	0.15	0.12	0.12	0.14	0.15	0.13	0.14	0.14	0.20
Psych Dept.	0.23	0.22	0.21	0.18	0.15	0.14	0.13	0.12	0.12	0.11	0.11	0.10	0.11	0.12	0.11	0.10
ID & TB	0.06	0.06	0.06	0.05	0.05	0.05	0.05	0.05	0.05	0.06	0.08	0.07	0.06	0.05	0.07	0.07
Onc Dept.	0.10	0.09	0.08	0.08	0.08	0.08	0.07	0.07	0.08	0.07	0.07	0.07	0.07	0.07	0.05	0.06
Rehab Dept.	0.16	0.13	0.13	0.13	0.12	0.12	0.11	0.10	0.11	0.10	0.09	0.08	0.08	0.08	0.08	0.09
TCM & Emd.	0.03	0.02	0.02	0.02	0.02	0.03	0.03	0.03	0.03	0.03	0.04	0.03	0.04	0.04	0.04	0.04
ITCWM Dept.	0.22	0.19	0.18	0.21	0.20	0.19	0.18	0.22	0.22	0.20	0.20	0.18	0.20	0.20	0.18	0.19
Other Depts.	0.09	0.09	0.08	0.15	0.12	0.10	0.11	0.10	0.09	0.08	0.08	0.09	0.04	0.03	0.03	0.04

**Table 6:** Interprovincial inequality in bed turnover by department

Departments	2009	2010	2011	2012	2013	2014	2015	2016	2017	2018	2019	2020	2021	2022	2023	2024
Overall	0.01	0.01	0.01	0.01	0.01	0.01	0.01	0.01	0.01	0.01	0.01	0.01	0.01	0.01	0.01	0.01
PMD & GMD	0.07	0.08	0.09	0.13	0.13	0.08	0.11	0.08	0.09	0.07	0.06	0.06	0.04	0.05	0.03	0.04
IM Dept.	0.01	0.01	0.01	0.01	0.01	0.01	0.01	0.01	0.01	0.01	0.01	0.02	0.01	0.02	0.01	0.01
Surg Dept.	0.01	0.01	0.01	0.01	0.01	0.01	0.01	0.01	0.01	0.01	0.01	0.02	0.02	0.02	0.02	0.02
Peds Dept.	0.03	0.02	0.02	0.02	0.02	0.02	0.01	0.01	0.02	0.02	0.01	0.03	0.04	0.03	0.02	0.01
OB/GYN Dept.	0.02	0.02	0.02	0.02	0.02	0.02	0.02	0.02	0.02	0.02	0.03	0.03	0.03	0.03	0.03	0.03
ENT-Ophth-Stom	0.03	0.03	0.03	0.02	0.02	0.02	0.03	0.03	0.03	0.03	0.02	0.04	0.04	0.03	0.03	0.02
Derm Dept.	0.10	0.08	0.09	0.07	0.08	0.08	0.07	0.03	0.04	0.04	0.05	0.06	0.07	0.07	0.05	0.04
AMD	0.15	0.25	0.18	0.23	0.21	0.23	0.20	0.20	0.21	0.22	0.22	0.40	0.42	0.42	0.18	0.30
Psych Dept.	0.10	0.10	0.08	0.08	0.11	0.13	0.14	0.08	0.10	0.08	0.09	0.06	0.06	0.05	0.05	0.05
ID & TB	0.03	0.03	0.03	0.03	0.03	0.03	0.03	0.03	0.04	0.04	0.05	0.07	0.09	0.07	0.05	0.06
Onc Dept.	0.02	0.01	0.01	0.01	0.01	0.01	0.01	0.01	0.01	0.02	0.01	0.02	0.02	0.02	0.02	0.03
Rehab Dept.	0.13	0.11	0.08	0.09	0.09	0.07	0.21	0.05	0.07	0.04	0.05	0.05	0.04	0.05	0.05	0.05
TCM & Emd.	0.02	0.02	0.02	0.02	0.02	0.01	0.02	0.02	0.02	0.02	0.02	0.03	0.03	0.03	0.02	0.02
ITCWM Dept.	0.07	0.07	0.06	0.06	0.06	0.03	0.02	0.02	0.02	0.02	0.03	0.04	0.04	0.04	0.04	0.04
Other Depts.	0.02	0.02	0.02	0.03	0.01	0.01	0.01	0.01	0.01	0.02	0.02	0.05	0.03	0.02	0.03	0.03

#### Within-province inequality across departments

Within provinces, interdepartmental inequality in bed supply remained consistently and substantially greater than that in bed turnover rates. The national GE(1) index for bed supply increased from 0.55 in 2009 to 0.58 in 2012–2013 and subsequently declined, with fluctuations, to 0.49 in 2024. In most provincial-level regions, the index was lower in 2024 than in 2009, with relatively large decreases in Beijing, Guangdong, Henan, Hebei, and Gansu. In 2024, the index remained relatively high in Tibet, Shaanxi, Inner Mongolia, and Heilongjiang, whereas Ningxia was among the few regions where inequality increased, from 0.51 to 0.58 (Table 7).

For bed turnover rates, the national GE(1) index decreased from 0.10 in 2009 to 0.09 in 2024 and showed relatively little variation over the study period. Based on the unrounded values, the index decreased in 15 provincial-level regions and increased in 16. The largest decreases occurred in Tibet, Yunnan, and Qinghai, while the largest increases occurred in Shanghai, Tianjin, and Anhui (Table 8).

**Table 7:** Within-province inequality in hospital bed supply across departments

Region	2009	2010	2011	2012	2013	2014	2015	2016	2017	2018	2019	2020	2021	2022	2023	2024
National	0.55	0.56	0.56	0.58	0.58	0.57	0.56	0.55	0.54	0.53	0.53	0.50	0.46	0.49	0.48	0.49
Beijing	0.55	0.60	0.58	0.53	0.51	0.52	0.51	0.48	0.47	0.47	0.47	0.45	0.41	0.43	0.45	0.44
Tianjin	0.56	0.56	0.55	0.55	0.55	0.56	0.56	0.53	0.57	0.63	0.62	0.52	0.51	0.51	0.56	0.59
Hebei	0.64	0.63	0.64	0.66	0.67	0.67	0.67	0.66	0.66	0.64	0.62	0.57	0.52	0.55	0.53	0.53
Shanxi	0.54	0.54	0.57	0.60	0.58	0.59	0.59	0.58	0.59	0.59	0.58	0.55	0.52	0.54	0.54	0.53
Inner Mongolia	0.59	0.61	0.61	0.63	0.61	0.61	0.61	0.61	0.58	0.56	0.57	0.56	0.55	0.56	0.55	0.59
Liaoning	0.58	0.57	0.58	0.62	0.62	0.61	0.59	0.58	0.58	0.58	0.58	0.56	0.53	0.56	0.56	0.55
Jilin	0.55	0.56	0.55	0.60	0.57	0.59	0.57	0.55	0.57	0.55	0.58	0.55	0.51	0.54	0.54	0.58
Heilongjiang	0.64	0.63	0.62	0.65	0.66	0.66	0.68	0.68	0.69	0.66	0.66	0.63	0.57	0.61	0.61	0.60
Shanghai	0.61	0.57	0.59	0.57	0.54	0.53	0.55	0.53	0.53	0.51	0.52	0.52	0.50	0.56	0.54	0.56
Jiangsu	0.57	0.59	0.58	0.58	0.57	0.55	0.55	0.55	0.55	0.53	0.54	0.52	0.49	0.51	0.51	0.50
Zhejiang	0.53	0.53	0.52	0.58	0.56	0.54	0.52	0.50	0.50	0.48	0.48	0.48	0.45	0.47	0.46	0.48
Anhui	0.52	0.52	0.52	0.55	0.54	0.52	0.52	0.52	0.50	0.48	0.48	0.46	0.42	0.44	0.43	0.44
Fujian	0.58	0.62	0.61	0.65	0.65	0.63	0.62	0.61	0.61	0.61	0.61	0.56	0.50	0.53	0.53	0.50
Jiangxi	0.56	0.57	0.56	0.58	0.60	0.57	0.57	0.56	0.53	0.52	0.51	0.49	0.46	0.47	0.47	0.47
Shandong	0.59	0.62	0.61	0.63	0.63	0.62	0.61	0.59	0.56	0.55	0.55	0.53	0.48	0.50	0.48	0.49
Henan	0.61	0.60	0.61	0.64	0.62	0.62	0.61	0.60	0.59	0.57	0.55	0.52	0.49	0.51	0.50	0.49
Hubei	0.54	0.55	0.54	0.55	0.53	0.52	0.52	0.51	0.50	0.52	0.51	0.48	0.45	0.47	0.47	0.47
Hunan	0.53	0.55	0.55	0.58	0.58	0.58	0.56	0.56	0.55	0.53	0.52	0.51	0.47	0.47	0.47	0.49
Guangdong	0.59	0.60	0.61	0.65	0.63	0.63	0.62	0.59	0.57	0.56	0.56	0.50	0.45	0.47	0.47	0.48
Guangxi	0.49	0.50	0.50	0.55	0.57	0.57	0.54	0.54	0.53	0.50	0.49	0.47	0.44	0.47	0.48	0.49
Hainan	0.52	0.54	0.51	0.52	0.49	0.49	0.49	0.46	0.48	0.47	0.45	0.43	0.40	0.42	0.41	0.44
Chongqing	0.54	0.55	0.55	0.57	0.57	0.57	0.57	0.54	0.53	0.51	0.50	0.50	0.46	0.50	0.51	0.54
Sichuan	0.54	0.54	0.54	0.57	0.56	0.55	0.54	0.52	0.52	0.52	0.52	0.52	0.49	0.51	0.52	0.53
Guizhou	0.57	0.58	0.58	0.60	0.61	0.60	0.58	0.53	0.54	0.52	0.51	0.50	0.47	0.49	0.49	0.49
Yunnan	0.58	0.58	0.58	0.61	0.61	0.62	0.61	0.61	0.61	0.61	0.59	0.54	0.52	0.55	0.53	0.54
Tibet	0.70	0.68	0.69	0.70	0.67	0.65	0.68	0.68	0.70	0.69	0.70	0.72	0.83	0.81	0.74	0.68
Shaanxi	0.68	0.71	0.71	0.76	0.75	0.76	0.75	0.74	0.71	0.71	0.71	0.64	0.57	0.60	0.59	0.61
Gansu	0.68	0.67	0.67	0.69	0.67	0.66	0.67	0.66	0.65	0.62	0.64	0.59	0.51	0.56	0.56	0.55
Qinghai	0.63	0.63	0.63	0.64	0.64	0.64	0.63	0.63	0.62	0.61	0.60	0.58	0.55	0.59	0.57	0.55
Ningxia	0.51	0.55	0.55	0.60	0.60	0.61	0.60	0.59	0.61	0.61	0.60	0.57	0.54	0.54	0.54	0.58
Xinjiang	0.57	0.56	0.57	0.58	0.57	0.56	0.56	0.56	0.54	0.54	0.50	0.54	0.50	0.52	0.51	0.50

**Table 8:** Within-province inequality in bed turnover across departments

Region	2009	2010	2011	2012	2013	2014	2015	2016	2017	2018	2019	2020	2021	2022	2023	2024
National	0.10	0.10	0.09	0.09	0.09	0.09	0.08	0.09	0.08	0.08	0.08	0.08	0.10	0.09	0.09	0.09
Beijing	0.20	0.19	0.19	0.24	0.23	0.17	0.20	0.20	0.23	0.21	0.20	0.20	0.21	0.18	0.17	0.17
Tianjin	0.13	0.14	0.19	0.18	0.17	0.18	0.14	0.18	0.18	0.19	0.20	0.23	0.23	0.20	0.16	0.18
Hebei	0.14	0.13	0.12	0.14	0.12	0.13	0.10	0.11	0.10	0.09	0.08	0.07	0.10	0.10	0.10	0.11
Shanxi	0.14	0.14	0.14	0.14	0.12	0.12	0.10	0.12	0.10	0.10	0.11	0.11	0.13	0.11	0.10	0.16
Inner Mongolia	0.10	0.13	0.14	0.12	0.14	0.18	0.15	0.17	0.15	0.15	0.16	0.16	0.18	0.16	0.14	0.11
Liaoning	0.11	0.11	0.09	0.11	0.13	0.13	0.12	0.12	0.11	0.11	0.13	0.11	0.14	0.14	0.13	0.15
Jilin	0.15	0.15	0.13	0.13	0.11	0.09	0.09	0.08	0.08	0.09	0.08	0.08	0.11	0.11	0.12	0.16
Heilongjiang	0.10	0.08	0.09	0.11	0.14	0.13	0.13	0.14	0.10	0.08	0.07	0.08	0.10	0.11	0.11	0.13
Shanghai	0.15	0.17	0.18	0.19	0.18	0.17	0.19	0.21	0.20	0.20	0.17	0.18	0.21	0.19	0.21	0.22
Jiangsu	0.12	0.09	0.09	0.11	0.12	0.12	0.11	0.12	0.09	0.11	0.12	0.11	0.13	0.12	0.13	0.14
Zhejiang	0.11	0.11	0.12	0.12	0.12	0.12	0.11	0.11	0.11	0.11	0.10	0.09	0.13	0.12	0.12	0.13
Anhui	0.09	0.11	0.09	0.10	0.10	0.10	0.10	0.10	0.08	0.08	0.08	0.09	0.12	0.11	0.11	0.14
Fujian	0.17	0.15	0.13	0.10	0.10	0.10	0.10	0.11	0.10	0.10	0.10	0.13	0.13	0.14	0.13	0.17
Jiangxi	0.10	0.12	0.11	0.14	0.12	0.10	0.10	0.10	0.11	0.11	0.09	0.10	0.10	0.10	0.12	0.12
Shandong	0.15	0.12	0.12	0.13	0.11	0.11	0.10	0.11	0.11	0.10	0.10	0.10	0.13	0.11	0.11	0.12
Henan	0.10	0.09	0.09	0.07	0.08	0.08	0.06	0.07	0.07	0.06	0.06	0.06	0.08	0.07	0.07	0.08
Hubei	0.10	0.09	0.09	0.08	0.07	0.07	0.06	0.07	0.07	0.07	0.07	0.07	0.09	0.08	0.09	0.11
Hunan	0.12	0.11	0.09	0.09	0.09	0.08	0.08	0.08	0.07	0.07	0.09	0.07	0.09	0.09	0.11	0.12
Guangdong	0.14	0.13	0.13	0.13	0.12	0.11	0.11	0.13	0.13	0.13	0.13	0.12	0.14	0.13	0.12	0.12
Guangxi	0.15	0.13	0.12	0.11	0.09	0.11	0.11	0.11	0.11	0.10	0.11	0.10	0.12	0.10	0.11	0.13
Hainan	0.12	0.12	0.11	0.13	0.12	0.12	0.12	0.10	0.10	0.09	0.11	0.10	0.13	0.14	0.13	0.12
Chongqing	0.11	0.10	0.09	0.08	0.08	0.08	0.07	0.07	0.07	0.07	0.07	0.07	0.09	0.08	0.09	0.10
Sichuan	0.12	0.10	0.08	0.08	0.09	0.08	0.09	0.10	0.08	0.08	0.07	0.07	0.09	0.07	0.08	0.09
Guizhou	0.10	0.08	0.08	0.07	0.06	0.07	0.06	0.07	0.08	0.08	0.07	0.07	0.11	0.09	0.09	0.09
Yunnan	0.13	0.11	0.09	0.08	0.07	0.07	0.08	0.09	0.07	0.08	0.07	0.06	0.09	0.08	0.08	0.08
Tibet	0.40	0.42	0.32	0.28	0.32	0.18	0.30	0.04	0.04	0.04	0.06	0.32	0.43	0.38	0.13	0.12
Shaanxi	0.09	0.08	0.10	0.10	0.09	0.09	0.07	0.07	0.07	0.06	0.07	0.06	0.09	0.09	0.08	0.09
Gansu	0.09	0.12	0.08	0.09	0.08	0.07	0.07	0.06	0.07	0.07	0.08	0.09	0.10	0.08	0.07	0.10
Qinghai	0.20	0.18	0.16	0.17	0.17	0.17	0.14	0.16	0.13	0.14	0.14	0.13	0.13	0.13	0.14	0.15
Ningxia	0.16	0.19	0.10	0.12	0.10	0.09	0.11	0.13	0.14	0.14	0.14	0.13	0.14	0.14	0.12	0.19
Xinjiang	0.09	0.09	0.08	0.07	0.07	0.07	0.08	0.07	0.06	0.05	0.07	0.09	0.10	0.09	0.08	0.12

## Discussion

This study identified two contrasting patterns of divergence between hospital bed supply and utilization across regions and departments in China. The first was a “low-supply–high-utilization” pattern. Bed supply grew more slowly in Beijing and Tianjin, whereas bed turnover rates remained high (Figs. 2 and 3). As regional medical centers, both cities receive substantial cross-provincial patient inflows, while urban space and planning constraints may limit bed expansion. A similar pattern was observed in oncology. In 2024, oncology had 1.97 beds per 10,000 population, a lower-middle level among departments, but the highest turnover rate, at 52.40 discharges per bed. Rising cancer hospitalization rates, together with declining in-hospital mortality and shorter hospital stays [42, 43], suggest that this high turnover may reflect increasing demand, faster patient throughput and, potentially, improvements in oncology care.

The second was a “high-supply–low-utilization” pattern. Bed supply increased markedly in the northeastern provinces of Liaoning, Jilin and Heilongjiang, but turnover rates remained low. The resident population of Northeast China has declined in recent years [44]. The shrinking population denominator may amplify the increase in beds per 10,000 population, while the reduction in the population served may constrain growth in inpatient service volume. In July 2026, the State Council issued the National Health Plan for the 15th Five-Year Plan Period, strengthening guidance and management of public hospital bed allocation [45]. This reflects a policy shift towards tighter capacity planning. At the department level, low turnover in psychiatry and rehabilitation may indicate that bed expansion has outpaced the development of complementary services. Turnover in these departments is strongly influenced by length of stay and service model [46, 47], while discharge often depends on community, family and follow-up care. Additional beds alone may therefore not translate directly into sustainable service capacity.

Kernel density and Markov chain analyses further indicated that bed supply grew more steadily than bed utilization, with divergent evolutionary trajectories across clinical departments. The main density peak for beds per 10,000 population shifted to the right in most departments, indicating broad growth in supply. Obstetrics and gynecology was the only department in which the peak initially shifted right and subsequently left, consistent with changes in family-planning policy and fertility levels [48, 49]. Bed supply increased particularly rapidly in internal medicine, surgery and traditional medicine, potentially reflecting health-system development, policies supporting traditional medicine, population ageing and the growing burden of chronic disease [50]. By contrast, density peaks for bed turnover shifted only slightly, indicating limited growth in utilization. Markov chain results showed that provincial bed-supply states generally remained unchanged or moved between adjacent levels, with few transitions across non-adjacent states. High-utilization states were particularly persistent: the probabilities of remaining at a high level were 0.90 for internal medicine and 0.88 for oncology (Table 4). Such persistence may reflect accumulated clinical capacity, staffing and regional diagnostic capacity, which cannot be readily redistributed through short-term changes in bed numbers [51].

At the interprovincial level, disparities in overall bed supply were small but remained substantial in several departments (Table 5). This suggests that convergence in total bed supply may mask persistent disparities across departments, as inequalities in obstetrics and gynecology, infectious diseases, and traditional medicine increased over the study period. The small overall disparity is consistent with previous estimates based on the Theil index and related methods [52], as well as China’s long-standing policy objective of promoting a more balanced regional distribution of health resources. From the patient perspective, however, a region may meet its overall bed target while lower relative supply may persist in specific departments. Whether this pattern limits access requires additional evidence on service needs, patient flows, waiting times, and health outcomes.

Within provinces, interdepartmental disparities in bed supply were substantially greater than those in bed turnover rates (Tables 7 and 8). Previous studies have likewise shown that health-resource allocation and service utilization exhibit different spatial patterns, and that the relationships among healthcare expenditure, service utilization, and quality display clear spatial heterogeneity; aggregate averages may obscure differences across regions and service types [53, 54]. Bed supply largely reflects planning decisions and established resource distributions, whereas utilization also depends on departmental service capacity, population health needs, and patients’ healthcare-seeking behavior [55, 56]. The decline in the bed-supply GE(1) index across most provinces in 2021 may reflect the temporary reallocation of hospital resources and disruption of routine services during the COVID-19 pandemic, which compressed interdepartmental differences. It should therefore not be interpreted as a lasting improvement in equality. The temporary increase in interprovincial inequality in AMD bed turnover rates during 2020–2022 may be related to the uneven recovery of elective and non-essential healthcare services during the COVID-19 pandemic. Because medical aesthetics services are more sensitive to restrictions on elective procedures and patient mobility, regional differences in service recovery may have temporarily widened disparities. A narrowing gap in bed supply across departments should therefore not be interpreted as improved allocation without considering utilization and service needs. In line with current policy priorities, hospital planning should combine aggregate capacity control with department-specific adjustment and should consider observed supply and utilization patterns together with independent assessments of population health needs and service capacity.

### Strengths and limitations

This study has four main strengths. First, leveraging official health statistics, we constructed a province–department–year panel dataset spanning 31 provincial-level administrative regions and 15 clinical departments in mainland China over the period 2009–2024. This dataset enabled a systematic assessment of long-term changes in hospital bed supply and utilization across regions and departments. Second, spatial analysis, kernel density estimation, and Markov chain analysis provided complementary evidence on distributional dynamics and the persistence of bed-allocation patterns. Third, jointly examining supply and utilization highlighted two contrasting patterns: low supply with high utilization and high supply with low utilization. This joint perspective goes beyond analyses of either dimension in isolation. Fourth, GE(1) quantified interprovincial disparities within departments and interdepartmental disparities within provinces, providing a multilevel assessment of inequalities in bed supply and utilization.

Several limitations should be noted. First, provincial-level official statistics provide good continuity and comparability and are suitable for characterizing national and interprovincial patterns. However, they do not capture cross-provincial patient flows or differences among health-care facilities within provinces. Second, bed supply was standardized by population, and utilization was measured using the bed turnover rate, without further adjustment for age structure or disease burden. Accordingly, the GE(1) results describe relative distributional differences rather than need-adjusted equity. Finally, this study focused on describing the distribution and evolution of bed supply and utilization. Future research could incorporate policy implementation and demographic change to investigate the underlying mechanisms.

## Conclusions

Between 2009 and 2024, hospital bed supply in China increased substantially, while interprovincial disparities in overall supply narrowed markedly. However, bed turnover rates increased only modestly, and supply and utilization followed different regional and departmental trajectories. High bed-supply states were persistent, and provincial transitions in both indicators occurred mainly between adjacent levels. Aggregate regional convergence did not eliminate persistent disparities across departments. Interprovincial disparities remained substantial in several departments and increased in obstetrics and gynecology, infectious diseases, and traditional medicine. Within provinces, interdepartmental disparities in bed supply remained substantially greater than those in bed turnover rates. Hospital bed planning should therefore move beyond aggregate capacity and interprovincial balance. It should manage growth in bed capacity, monitor department-specific supply and utilization, and reallocate existing beds according to population health needs and departmental service capacity. This would shift planning from expanding bed numbers to optimizing their distribution across departments.

## Data Availability

The datasets used in this study were derived from published statistical yearbooks. The processed data supporting the findings of this study are available from the corresponding author upon reasonable request.
